# Associations between Biomarkers of Metal Exposure and Dry Eye Metrics in Shipyard Welders: A Cross-Sectional Study

**DOI:** 10.3390/ijerph19042264

**Published:** 2022-02-17

**Authors:** Ying-Hsi Liou, Ying-Jen Chen, Wei-Liang Chen, Kuan-Ying Li, Ting-Yu Chou, Yung-Chi Huang, Chung-Ching Wang, Ching-Huang Lai

**Affiliations:** 1 School of Medicine, National Defense Medical Center, Taipei 114, Taiwan; philip27941137@mail.ndmctsgh.edu.tw (Y.-H.L.); m871079@mail.ndmctsgh.edu.tw (W.-L.C.); bigching@gmail.com (C.-C.W.); 2 Department of Ophthalmology, Tri-Service General Hospital, Taipei 114, Taiwan; yj12664@gmail.com; 3 Division of Family Medicine, Department of Family and Community Medicine, Tri-Service General Hospital, Taipei 114, Taiwan; 4 Division of Geriatric Medicine, Department of Family and Community Medicine, Tri-Service General Hospital, Taipei 114, Taiwan; 5 Department of Public Health, School of Public Health, National Defense Medical Center, 161 Min-Chuan E. Rd., Sec. 6, Taipei 114, Taiwan; handsomeian89@gmail.com (K.-Y.L.); d0937890755@gmail.com (T.-Y.C.); zxc95103@gmail.com (Y.-C.H.)

**Keywords:** PM_2.5_, metals, welding fume, dry eye

## Abstract

Shipyard welders are often exposed to welding metal fumes. Ocular surfaces are continually exposed to environmental hazards. However, limited information on the associations between metal exposure and dry eye metrics in occupational settings is available. This study employed a cross-sectional design that involved the participation of 59 welders and 25 administrative staff in a shipyard in northern Taiwan from September 2020 to October 2020. The participants’ individual information, laboratory data, exposure to particulate matter < 2.5 μm, urinary, and toenail metal concentrations were collected. Dry eye metrics were evaluated using standardized questionnaires and a noninvasive ocular surface analyzer. Urinary V and Cr and toenail V, Cr, Mn, Fe, Ni, Zn, As, and Cd and Pb were significantly higher in the exposed group than in the control group. After adjustment for confounding factors, dry eye metrics were associated with urinary Cd (β = 0.407; *p* = 0.007) and toenail Pb (β = 0.482; *p* = 0.002). The participants with higher urinary Cd exhibited higher odds ratios for elevated dry eye metrics. Our study revealed that exposure to welding procedures increases several metal biomarkers. In addition, urinary Cd, and toenail Pb might be related to dry eye disease in shipyard welders.

## 1. Introduction

An estimated 11 million people are employed as welding workers worldwide. Welding procedures may involve exposure to metal fumes, gas, and ultraviolet radiation. Welding fumes contain fine particulate matter and several heavy metals on which welding materials and processes depend. The International Agency for Research on Cancer classified welding fumes as “carcinogenic to humans” (Group 1) in 2017 [1]. In addition, several studies have indicated that exposure to welding fumes might be associated with increased inflammation [2], advanced glycation end-products [3], and risk of cardiovascular disease [4].

Dry eye disease (DED) is a common ocular surface disorder. Common symptoms of DED are ocular pain, dryness, irritation, and fluctuating vision [5]. DED prevalence among adults in the United States is approximately 6.8% (16.4 million people) in adults [6]. The annual economic burden of DED on the United States health care system is about US$3.8 billion annually [7]. The common causes of DED are inadequate secretion of tears and tear fluid evaporation [5]. The risk factors for dry eye are contact lens use; medications, such as antihistamines and antidepressants; and systemic diseases, such as Sjögren’s syndrome [8]. In addition, several studies have investigated the relationship between environmental factors (including high wind velocity, high airborne particulates, low humidity, and high temperature) and dry eye, including high wind velocity, high airborne particulates, low humidity, and high temperature [5,9].

The retina and optic nerve can absorb toxic metals from the blood through the choriocapillaris, contributing to age-related macular degeneration [10]. In addition, patients with mercury intoxication may present neurogenic-based ocular surface disorders [11]. According to a population-based study, plasma mercury levels are positively associated with dry eye symptoms [12]. However, these studies investigated the association between plasma metals and DED only by observing the participants’ dry eye symptoms without using clinical questionnaires or an objective evaluation index [12,13]. Moreover, little is known about the relationship between metal biomarkers and DED in occupational settings.

Our study analyzed the association between urinary and toenail metal concentrations and dry eye metrics. We hypothesized that individuals with greater metal exposure would exhibit more severe signs and symptoms of dry eye.

## 2. Materials and Methods

### 2.1. Study Design

Our cross-sectional study was conducted in a shipyard in northern Taiwan from September 2020 to October 2020. The Institutional Review Board approved the study of the Tri-Service General Hospital in Taipei, Taiwan. The inclusion criteria were age above 20 and employment at the shipyard company. The exclusion criteria were as follows: (a) a history of Sjögren’s syndrome, (b) contact lens use, (c) a history of laser refractive surgery, (d) use of ocular medications or artificial tears, and (e) use of medications that may reduce tear production.

### 2.2. Study Population

Sixty-two welders and 27 office workers were invited to our study. There were 59 welders (exposed group) and 25 office workers (control group) who participated in this current study. Three welders and two office workers were excluded because of incomplete specimens’ collection. Therefore, none of the invited participants meet the exclusion criteria above.

### 2.3. Data Collection Procedures

The participants were asked to wear PM_2.5_ samplers during their working hours to determine their 8-h PM_2.5_ exposure on the first weekday after study commencement. Basic individual parameters, including the participants’ living habits, work status, and dietary habits, were also collected by a questionnaire. The following morning, urine, toenails, and blood samples were collected from the participants. The participants’ dry eye metrics were evaluated using dry eye questionnaires and a noninvasive ocular surface analyzer (ICP OSA; SBM Sistemi, Orbassano, Italy).

### 2.4. Personal Information and Dry Eye Questionnaires

Demographic data, including age, body mass index (BMI), smoking habits, drinking habits, and medical history, were collected for each participant. We used two standardized dry eye questionnaires, the ocular surface disease index (OSDI) and the standardized patient evaluation of eye dryness (SPEED), to determine the severity of each participant’s dry eye symptoms.

OSDI scores range from 0 to 100; scores < 13 indicate standard values, and higher scores indicate more severe dry eye symptoms [14]. SPEED scores range from 0 to 28, with higher scores indicating more severe dry eye symptoms [15].

### 2.5. Environmental Factor Monitoring

The 8-h PM_2.5_ exposure of each participant was calculated using a personal PM_2.5_ sampler (224-PCXR pump, SKC, Eighty-Four, PA, USA) in the welding or administration area. The filters were conditioned for at least 24 h and subsequently passed over a static neutralizer (Allfield, Taipei, Taiwan) to reduce the electrostatic charge before weighing.

Hourly temperature and relative humidity data were collected from the local central weather bureau station during the sampling period.

### 2.6. Biochemistry Test

After 8-h overnight fasting, venous blood samples were collected in BD Vacutainer tubes (Becton Dickinson, Rutherford, NJ, USA). Serum-separating tubes, ethylenediaminetetraacetic acid tubes, and fluoride tubes for biochemical, routine, and insulin tests.

### 2.7. Toenail and Urinary Metal Concentration Measurements

The participants were instructed not to clip their toenails for one week before sample collection to ensure that adequate toenail samples could be collected from all ten toes on the collection date. The 50-mg toenail samples were weighed, washed by sonication using acetone, oven-dried, and digested in nitric acid diluted with deionized water.

Urine samples were collected in 50-mL polypropylene conical tubes (Falcon, Corning Science, Reynosa, Tamaulipas, Mexico) and digested in nitric acid.

We used inductively coupled plasma mass spectrometry (ICP-MS, Thermo Fisher, San Francisco, CA, USA) to detect the concentrations of vanadium (V), chromium (Cr), manganese (Mn), iron (Fe), nickel (Ni), cobalt (Co), copper (Cu), zinc (Zn), arsenic (As), selenium (Se), cadmium (Cd), mercury (Hg), and lead (Pb) in the toenail and urine samples. The analyzes was repeated twice.

The recovery efficiencies of the elements from the urine samples were determined by spiking a known quantity of a trace element (NIST SRM 2670a) into a urine sample and following the same experimental procedure used to treat the urine samples initially. The recovery efficiencies were as follows: V, 102%; Cr, 99%; Mn, 103%; Fe, 105%; Co, 105%; Ni, 105%; Cu, 102%; Zn, 94%; As, 104%; Cd, 104%; and Pb, 104%. The blank tests were performed using the same procedure used in the recovery efficiency tests, but without adding the known standard solution. The limits of detection (in μg/L) were as follows: V, 0.028; Mn, 0.027; Co, 0.004; Cu, 0.025; Zn, 0.075; As, 0.027; Cd, 0.017; Ni, 0.032; and Cr, 0.037. At these limits, the signal-to-noise ratio was 3.

The analyzes of the blanks, including field blanks and lab blanks, revealed no significant contamination (i.e., the ICP-MS integrated area was below the detection limit). All sample preparation and measurement steps were performed in a laminar flow cabinet.

### 2.8. Ocular Surface Analyzes (OSA)

A noninvasive OSA was used to evaluate the participants’ dry eye metrics. Namely, their tear meniscus heights (normal values are ≥0.2 mm; lower values indicate more severe dry eye signs), a cross-sectional metric of tear volume. In addition, Schirmer’s test (normal values are >10 mm, lower values indicate more severe dry eye signs) was performed with anesthesia to measure each participant’s tear volume with a paper strip applied over the lower eyelid. The value used for analyzes was from all tests conducted using each participant’s more severe dry eye.

### 2.9. Statistical Analysis

All statistical analyzes were conducted in SPSS version 22.0 (IBM). Because the urinary and toenail metal concentrations and dry eye metrics were not normally distributed, log transformation was used to obtain a normal distribution. The Mann–Whitney test was conducted to evaluate the differences between the exposed and control groups. Spearman correlation, multivariant linear regression, and logistic regression analyzes were conducted to determine the association between metal biomarkers and dry eye metrics. A *p* value of <0.05 indicated a significant difference.

## 3. Results

### 3.1. Characteristics of the Study Population

A comparison of the demographic data and dry eye metrics of the exposed group and the control group is presented in Table 1. The average cholesterol (*p* = 0.027) in the exposed group was significantly higher than those in the control group. The average age (*p* = 0.002) was significantly lower than the control group. No differences in BMI, fasting plasma glucose (FPG), hemoglobin A1c, PM_2.5_, temperature, humidity, OSDI, SPEED, Schirmer’s test scores, or tear meniscus height were identified between the two groups. The dry eye metrics in the exposed group were slightly lower than in the control group, but the difference was not significant.

### 3.2. Characteristics of Metal Biomarkers

Each group’s average urinary and toenail metal concentrations are listed in Table 2. Urinary V (*p* = 0.014) and Cr (*p* = 0.022) and toenail V (*p* = 0.002), Cr (*p* = 0.015), Mn (*p* = 0.000), Fe (*p* = 0.000), Ni (*p* = 0.004), Zn (*p* = 0.025), As (*p* = 0.035), Cd (*p* = 0.043), Pb (*p* = 0.018) were significantly higher in the exposed group than in the control group. Urinary creatinine showed no significant differences between two groups.

### 3.3. Correlations among Environmental Factors and Dry Eye Metrics

The correlational coefficients between several environmental factors and dry eye metrics are listed in Table 3. PM_2.5_ and temperature are not significantly related to dry eye metrics, and humidity was related considerably to tear meniscus height (ρ = −0.336, *p* < 0.05).

### 3.4. Correlations among Metal Biomarkers and Dry Eye Metrics

The correlation coefficients between urinary and toenail metal concentrations and dry eye metrics revealed several significant correlations, as indicated in Table 4. Urinary Co and Cd were significantly positively related to OSDI (ρ = 0.267, *p* < 0.05 and ρ = 0.298, *p* < 0.01, respectively) and SPEED (ρ = 0.226, *p* < 0.05 and ρ = 0.226, *p* < 0.05, respectively) scores. Toenail V and Ni were significantly related to tear meniscus height (ρ = 0.249, *p* < 0.05 and ρ = 0.249, *p* < 0.05, respectively).

### 3.5. Associations between Metal Biomarkers and Dry Eye Metrics

**Figure 1:**
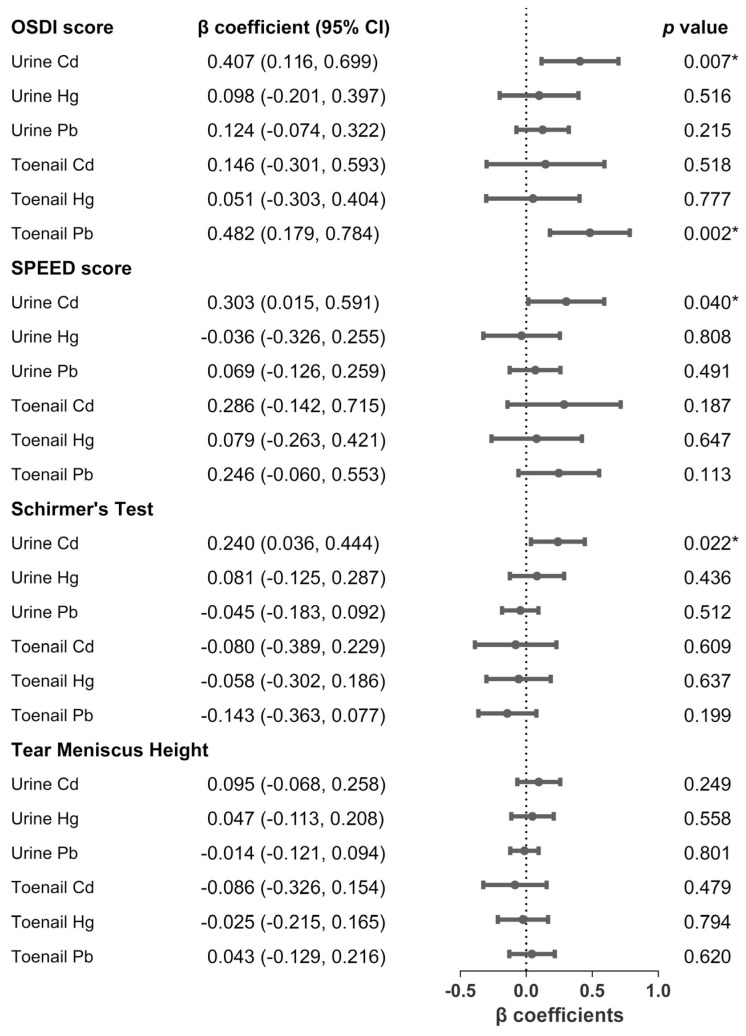
Association between urinary and toenail metals concentrations and dry eye metrics ^a^. * *p* < 0.05. ^a^ Data were adjusted for smoking and drinking habits, age, temperature, humidity, and fasting plasma glucose. Abbreviations: OSDI, ocular surface disease index; SPEED, standardized patient evaluation of eye dryness questionnaire; Cd, cadmium; Hg, mercury; Pb, lead.

We investigated the associations between three toxic metals (Cd, Hg, and Pb) and dry eye metrics through multivariate linear regression, as displayed in Figure 1. Urinary Cd was significantly associated with OSDI (β = 0.407; 95% CI, 0.116–0.699; *p* = 0.007), SPEED (β = 0.303; 95% CI, 0.015–0.591; *p* = 0.040), and Schirmer’s test (β = 0.240; 95% CI, 0.036–0.444; *p* = 0.022) scores after an adjustment for smoking and drinking habits, age, temperature, humidity, and FPG. Toenail Pb was significantly associated with the OSDI score (β = 0.482; 95% CI, 0.179–0.784; *p* = 0.002).

### 3.6. Associations between Urinary Cd and Ocular Surface Measurements

**Figure 2:**
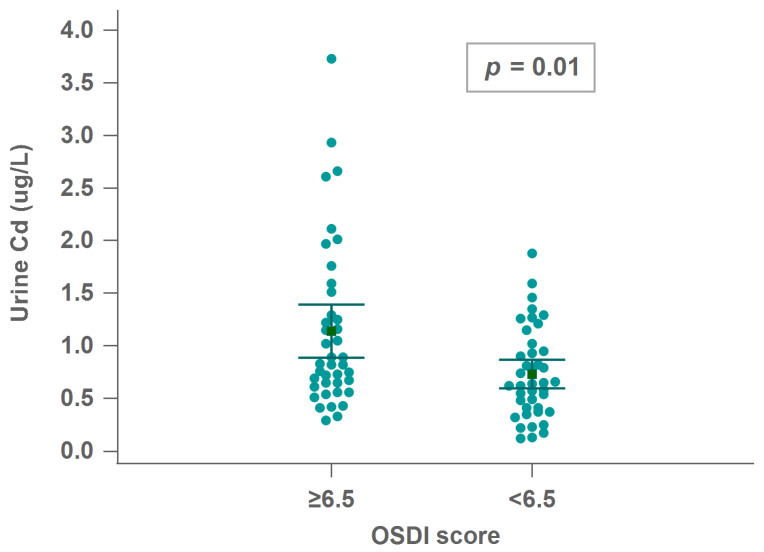
Multiple comparison graph of urinary Cd and OSDI scores. Abbreviations: OSDI, ocular surface disease index; Cd, cadmium.

The cutoff points (50th percentile) for OSDI and Schirmer’s test were 6.5 and 7 mm, respectively. Each 1-μg/L increase in urinary Cd concentration resulted in a 2.26-fold and 2.309-fold higher odds ratio of an OSDI score ≥ 6.5 in the univariate analysis and model 1 compared to an OSDI score < 6.5, respectively. However, the association was close to the cutoff point in models 2 and 3. There was no significant association between urinary Cd and low Schirmer’s test scores (Table 5). As indicated in Figure 2, urinary Cd was significantly higher in the high-OSDI group (*p* = 0.01).

## 4. Discussion

In this study, the exposed group (welding workers) exhibited significantly higher metal biomarkers concentrations than the control group (administrative staff), indicating that welding procedures resulted in greater metal exposure. In addition, our study noted that dry eye metrics were positively related to short-term Cd exposure and long-term Pb exposure after an adjustment for confounding factors.

Our preceding study reported that Fe, Zn, Mn, and Cu were dominant in the metal fume in the same shipyard, and welding workers had higher urinary levels of Cu, Ni, Mn, Cd, and Zn [4]. Notably, we observed that the PM_2.5_ levels were not significantly different in the exposed group than in the control group at this data collection time point. Most of the urinary metals were no different; only urinary levels of V and Cr were higher in the welders than in-office workers. We explained that the fluctuation in welding work was influenced mainly by the COVID-19 epidemic during our data collection period. The PM_2.5_ concentration of welders was dramatically decreased from 1013 μg/m^3^ to 288 μg/m^3^. In contrast, the levels of PM_2.5_ from administrative workers were not significantly different from the previous study [18].

Cholesterol and age were significantly different in the exposed group and control group. During the KNHANES (Korea National Health and Nutrition Examination Survey) V, performed from 2010 to 2011, a dry eye questionnaire survey was conducted. Dry eye disease in the Korean women population was associated with high serum cholesterol [19]. Another study also found that the prevalence of hyperlipidemia was significantly higher in patients with dry eye disease [20]. This Taiwanese population-based study group comprised all patients who sought outpatient care diagnosed with dry eye disease (International Classification of Disease, 9th edition, Clinical Modification (ICD-9-CM) code 375.15, tear film insufficiency, unspecified). After adjusting confounders, such as gender, age, and socioeconomic status, they showed that patients with dry eye disease had a higher hyperlipidemia risk than the control group. The possible mechanism of hypercholesterolemia in dry eye disease could be proposed as increased cholesterol in the meibomian lipid. It would increase its melting point to 46 °C versus the usual meibomian lipid melting point of 30–34 °C and, thus, lead to increased viscosity and plugging of the meibomian orifice [21]. In addition, the lacrimal gland is significantly damaged with advanced age. Age-related eyelid changes include lid laxity, meibomian gland atrophy, orifice metaplasia, decreased tear volume, increased tear breakup time, and dry eye. Aging is often accompanied by cardiovascular disease, type 2 diabetes, depression, glaucoma, and other eye diseases. Some of these comorbidities themselves, or the mediations used to treat them, may have harmful secondary effects on the ocular surface [22].

Urinary metal concentrations reflect recent exposure (within several days), whereas toenail metal concentrations reflect cumulative exposure (within 6–12 months) [23,24,25,26]. Cd and Pb are ubiquitous pollutants that can cause acute and chronic toxicity: Cd is associated with kidney and bone dysfunction [27]. In contrast, Pb can cause neuropathies, kidney injury, and visual deterioration [28,29]. Similarly, Pb and Cd are responsible for the most common and dangerous forms of eye toxicity [30]. Both metals accumulate in human ocular tissues, especially in the retinal pigment epithelium and choroid [31]. In addition, Pb can produce oxidative stress and contribute to pathogenesis due to the increased production of reactive oxygen species (ROS) [32].

Lead (Pb) can absorb via the respiratory tract, gastrointestinal tract, and skin. In addition, lead is distributed to blood, bone, and soft tissue. Over 95% of circulating lead is bound to erythrocyte proteins [33]. Therefore, clinically, the blood lead level (BLL) is essential for diagnosing lead poisoning and treatment [34]. We suspected that urinary Pb was not related to dry eye metrics because lead is mainly distributed to erythrocyte in plasma rather than urine. Therefore, urinary Pb may not sufficiently represent the lead internal dose concentration.

Several studies have reported that DED is associated with low humidity [9]. By contrast, our study revealed that high humidity was related to more severe signs of dry eye. A possible explanation is that high moisture might increase particulate matter levels, thereby increasing the incidence of DED [35].

We hypothesize that metal exposure may cause dry eye symptoms by leading to an oxidative stress imbalance. Oxidative stress affects several eye structures, including the ocular surface, retina, and lens. ROSs may contribute to tear lipid layer damage and inflammation, which leads to dry eye symptoms [36]. In an animal study, oxidative stress was associated with ocular surface epithelial damage, contributing to DED [37]. In addition, decreased expression of antioxidant enzymes in the conjunctival epithelium increases the severity of dry eye symptoms [38].

In the present study, tungsten inert gas (TIG) was the welding process in the shipyard. TIG is a standard method for welding in various industrial sectors due to its solid and high-quality welds. Most metallic particles with nano-sized fractions are generated during the TIG welding processes, which becomes a primary occupational health concern. Mn, Si, Ni, Mo, and Cr were the significant elements determined in the welding rods used in the shipyard [39]. Therefore, PM_2.5_ exposure in welding workers can not be eliminated. Furthermore, all welding workers were requested to wear masks, which may lead to overestimating the exposure levels of PM_2.5_ in welding workers. Heavy metals intake from drink and food may have influenced the determination of urinary metals.

The prevalence of dry eye symptoms was 28.8% and 40% in exposed and reference groups, respectively. Compared to some studies that used the same dry eye questionnaire as diagnostic criteria, the prevalence of DED was 44.3% in undergraduate students who were aged 18–34, non-contact lens, and non-smokers in Ghana. The prevalence between different studies (ranged from 10% to 70.4) was somewhat different despite using the same OSDI questionnaire in similar college students. DED prevalence in the young Thai population was less than that reported in Thai adults (34%) using the woman’s health study questionnaire [40]. Participants’ demographics could directly explain the reason and diagnostic criteria used.

Bazeer et al. conducted a large population-based occupational study that used the women’s health study (WHS) dry eye questionnaire to assess the symptomatic dry eye [41]. After adjusting for age and sex, dry eye associated comorbidities, building workers and metal and machinery workers had the highest risk of dry eye. In addition, this study showed that people with indoor and sedentary occupations have a higher prevalence of dry eye disease. They also found a strong protective effect of most outdoor and physically active occupations on dry eye disease. The highest prevalence of dry eye was found in the clerical support workers and professionals of all occupations. These occupations are associated with relatively high visual display terminals (VDTs) and sedentary occupations. Furthermore, contact lens use was the most crucial confounder [42], however, the welders and office workers with no contact lenses were used in our study.

A proposed mechanism is that office workers’ physical inactivity and sedentary behavior leads to increased systemic inflammation via oxidative stress, including ocular surface inflammation and reduced mucin expression with subsequent increased tear film break up. The pathologic mechanisms of gaseous and particulate matter pollutants underlying disease may vary by air pollution type. Moreso, shared mechanisms include oxidative stress and inflammation induction with subsequent nerve, vascular, and DNA damage. This study implies that ergonomic measures can then be taken, such as humidifiers and screen protectors for VDTs in office environment strategies that may lower symptomatic dry eye. In addition, in welding workers, appropriate eyewear might prevent dry eyes.

A strength of our study was the use of two subjective personal DED questionnaires and three accurate ocular surface indices to determine the relationship between metal biomarkers and dry eye metrics. Moreover, this study used both short-term (urine) and long-term (nail) exposure indicators as metal biomarkers.

Our study still has some limitations. First, a cross-sectional study could not distinguish the temporal relationship between metal biomarkers and dry eye metrics cannot imply causality. Therefore, a longitudinal cohort study should be conducted in the future. Second, the generalizability of our results is limited for the sample size and male workers in this current study, instead of several shipyard welding industries. Third, no data was available for gaseous air pollutants at the sampling sites, including reactive gases, such as carbon monoxide, nitrogen dioxide, and volatile organic compounds, in this study. In addition, it focused on the effects of outdoor air pollution on dry eye health. However, indoor air pollution, environmental factors, such as air conditioning, low humidity, and visual display terminal use contribute to dry eye disease [43]. Furthermore, the healthy worker effect might be explained by the low prevalence of dry eye symptoms and signs, though we have adjusted the age confounder.

## 5. Conclusions

This cross-sectional study revealed that the concentrations of several metal biomarkers were significantly higher in the exposed group than in the control group. Urinary Cd and toenail Pb were determined to be associated with dry eye metrics in shipyard welders. This study may serve as a valuable reference for implementing preventive measures against occupational hazards. More research is necessary to investigate the relationships between metals exposure and dry eye metrics and the mechanisms underlying these relationships.

## Figures and Tables

**Table 1:** Descriptive statistics of the study population.

Variable	Total (*n* = 84)	Exposed Group (*n* = 59)	Control Group (*n* = 25)	*p* Value
Continuous variable (Mean ± SD)				
Age (years)	44.27 ± 12.75	41.15 ± 11.76	51.64 ± 12.16	0.002 *
BMI (kg/m^2^)	26.36 ± 3.93	26.14 ± 3.77	26.89 ± 4.32	0.463
FPG (mg/dL)	94.57 ± 18.83	94.83 ± 21.41	93.96 ± 10.86	0.573
Categorical variables (*n* (%))				
Smoking	25 (29.8)	19 (32.2)	6 (24.0)	0.455
Drinking	21 (25.0)	14 (23.7)	7 (28.0)	0.681
Cholesterol (mg/dL)	196.04 ± 50.36	202.05 ± 56.69	181.84 ± 26.54	0.027 *
HbA1c (%)	5.57 ± 0.80	5.61 ± 0.91	5.49 ± 0.42	0.616
PM_2.5_ (μg/m^3^)	286.05 ± 69.44	288.35 ± 73.54	280.63 ± 59.68	0.758
Temperature (°C)	25.54 ± 1.27	25.39 ± 1.26	25.91 ± 1.25	0.098
Humidity (%)	73.57 ± 16.52	75.61 ± 17.08	68.76 ± 14.32	0.092
Dry eye metrics (Mean ± SD) ^a^				
OSDI score ^b^	7.20 ± 5.85	6.98 ± 5.85	7.72 ± 5.95	0.596
SPEED score ^c^	4.75 ± 4.51	4.76 ± 4.90	4.72 ± 3.53	0.639
Schirmer’s Test (mm) ^d^	10.12 ± 7.94	10.27 ± 7.49	9.76 ± 9.08	0.277
Tear Meniscus Height (mm) ^e^	0.14 ± 0.07	0.14 ± 0.07	0.15 ± 0.08	0.825
Dry eye metrics (*n* (%))				
OSDI score ≥ 13 ^b^	18 (21.4)	12 (20.3)	6 (24.0)	0.710
SPEED score > 6 ^c^	27 (32.1)	17 (28.8)	10 (40.0)	0.318
OSDI score ≥ 13 and SPEED score > 6	15 (17.8)	10 (16.9)	5 (20.0)	0.740
Schirmer’s Test ≤ 10 mm ^d^	54 (64.2)	37 (63.7)	17 (68.0)	0.646
Tear Meniscus Height < 0.2 mm ^e^	71 (84.5)	50 (84.7)	21 (84.0)	0.932

* *p* < 0.05. Abbreviations: SD, standard deviation; BMI, body mass index; FPG, fasting plasma glucose; HbA1c, hemoglobin A1c; PM_2.5_, particulate matter ≤ 2.5 μm; OSDI, ocular surface disease index; SPEED, standardized patient evaluation of eye dryness questionnaire. ^a^ Higher OSDI and SPEED scores and lower Schirmer’s test scores and tear meniscus heights are associated with more severe dry eye symptoms. ^b^ Scores range from 0 to 100 (<13 indicates normal value) [14]. ^c^ Scores range from 0 to 28 (cutoff point of 6 is considered for dry eye symptoms) [15,16]. ^d^ >10 mm indicates normal value [14]. ^e^ ≥0.2 mm indicates normal value [17].

**Table 2:** Comparison of the metal biomarker concentrations between the exposed and control groups.

Variable	Total (*n* = 84)	Exposed Group (*n* = 59)	Control Group (*n* = 25)	*p* Value
Urinary creatinine (Mean ± SD)				
(mg/dL)	0.95 ± 0.24	0.93 ± 0.13	1.00 ± 0.039	0.747
Urinary metal level (Mean ± SD)				
V (μg/L)	0.49 ± 0.23	0.52 ± 0.22	0.42 ± 0.25	0.014 *
Cr (μg/L)	1.05 ± 0.66	1.14 ± 0.75	0.83 ± 0.26	0.022 *
Mn (μg/L)	0.81 ± 0.87	0.77 ± 0.79	0.88 ± 1.05	0.926
Fe (μg/L)	48.62 ± 26.33	48.86 ± 23.22	48.06 ± 33.06	0.335
Ni (μg/L)	1.55 ± 3.29	1.14 ± 2.01	2.53 ± 5.13	0.437
Co (μg/L)	0.24 ± 0.49	0.18 ± 0.49	0.38 ± 0.49	0.179
Cu (μg/L)	1.31 ± 3.07	1.36 ± 3.02	1.20 ± 3.24	0.114
Zn (μg/L)	886.28 ± 532.32	952.07 ± 545.23	731.01 ± 475.17	0.081
As (μg/L)	202.02 ± 361.28	215.27 ± 422.03	170.75 ± 139.72	0.788
Se (μg/L)	77.41 ± 33.57	81.44 ± 33.38	67.90 ± 32.73	0.670
Cd (μg/L)	1.08 ± 1.04	1.08 ± 1.14	1.08 ± 0.80	0.442
Hg (μg/L)	1.42 ± 0.98	1.40 ± 0.93	1.47 ± 1.11	0.872
Pb (μg/L)	0.12 ± 0.32	0.12 ± 0.33	0.12 ± 0.31	0.721
Toenail metal level (Mean ± SD)				
V (μg/g)	0.10 ± 0.60	0.13 ± 0.71	0.03 ± 0.04	0.002 *
Cr (μg/g)	4.56 ± 14.80	5.15 ± 17.13	3.17 ± 6.75	0.015 *
Mn (μg/g)	1.95 ± 4.70	2.46 ± 5.51	0.74 ± 0.91	0.000 *
Fe (μg/g)	120.90 ±273.76	147.81 ± 319.78	57.40 ± 77.86	0.000 *
Ni (μg/g)	0.04 ± 0.13	0.05 ± 0.15	0.02 ± 0.03	0.004 *
Co (μg/g)	4.46 ± 9.14	5.04 ± 10.46	3.11 ± 4.64	0.149
Cu (μg/g)	4.15 ± 8.78	4.64 ± 10.04	2.98 ± 4.60	0.263
Zn (μg/g)	8.74 ± 17.16	9.49 ± 19.71	6.98 ± 8.64	0.025 *
As (μg/g)	203.48 ± 494.20	229.76 ± 586.01	141.45 ± 95.12	0.035 *
Se (μg/g)	201.58 ± 491.96	227.38 ± 583.40	140.69 ± 94.81	0.053
Cd (μg/g)	201.87 ± 491.85	227.96 ± 583.26	140.28 ± 94.25	0.043 *
Hg (μg/g)	202.78 ± 495.73	229.00 ± 587.90	140.90 ± 94.49	0.060
Pb (μg/g)	0.29 ± 0.48	0.34 ± 0.56	0.19 ± 0.17	0.018 *

* *p* < 0.05. Abbreviations: SD, standard deviation; V, vanadium; Cr, chromium; Mn, manganese; Fe, iron; Ni, nickel; Co, cobalt; Cu, copper; Zn, zinc; As, arsenic; Se, selenium; Cd, cadmium; Hg, mercury; Pb, lead.

**Table 3:** Correlation coefficients among environment factors and dry eye metrics ^a^.

Variables	OSDI	SPEED	Schirmer’s Test	Tear Meniscus Height
PM_2.5_ (μg/m^3^)	0.103	0.028	0.009	−0.047
Temperature (°C)	0.069	−0.001	0.056	0.185
Humidity (%)	0.003	0.034	−0.096	−0.336 *

* *p* < 0.05. Abbreviations: OSDI, ocular surface disease index; SPEED, standardized patient evaluation of eye dryness questionnaire; PM_2.5_, particulate matter ≤ 2.5 μm. ^a^ Higher OSDI and SPEED scores and lower Schirmer’s test scores and tear meniscus heights are associated with more severe dry eye symptoms.

**Table 4:** Correlational coefficients among metal biomarkers and dry eye metrics ^a^.

Variables	OSDI	SPEED	Schirmer’s Test	Tear Meniscus Height
Urinary metal (μg/L)				
V	0.093	0.055	0.187	−0.032
Cr	0.089	0.028	0.112	−0.104
Mn	0.056	0.095	0.094	−0.011
Fe	0.058	0.034	0.102	−0.102
Ni	0.027	−0.017	−0.094	0.055
Co	0.267 *	0.226 *	0.005	0.083
Cu	0.185	0.135	0.077	−0.014
Zn	0.194	0.124	0.190	−0.005
As	0.058	0.016	−0.019	−0.061
Se	0.276 *	0.125	0.139	0.068
Cd	0.298 **	0.226 *	0.098	0.046
Hg	0.093	0.011	0.088	0.003
Pb	0.167	0.149	−0.043	−0.153
Toenail metal (μg/g)				
V	−0.012	−0.020	−0.040	0.249 *
Cr	−0.127	−0.125	0.007	0.057
Mn	−0.103	−0.134	0.007	0.154
Fe	−0.059	−0.050	−0.038	0.185
Ni	−0.052	−0.017	0.085	0.249 *
Co	0.179	0.100	0.080	0.105
Cu	0.210	0.126	0.072	0.103
Zn	−0.006	0.007	0.021	0.062
As	0.166	0.154	−0.063	−0.158
Se	0.166	0.136	−0.080	−0.122
Cd	0.172	0.146	−0.065	−0.139
Hg	0.175	0.158	−0.101	−0.114
Pb	0.239 *	0.178	−0.032	0.070

* *p* < 0.05; ** *p* < 0.01. Abbreviations: OSDI, ocular surface disease index; SPEED, standardized patient evaluation of eye dryness questionnaire; V, vanadium; Cr, chromium; Mn, manganese; Fe, iron; Ni, nickel; Co, cobalt; Cu, copper; Zn, zinc; As, arsenic; Se, selenium; Cd, cadmium; Hg, mercury; Pb, lead. ^a^ Higher OSDI and SPEED scores and lower Schirmer’s test scores and tear meniscus heights are associated with more severe dry eye symptoms.

**Table 5:** Association between urinary Cd and high-OSDI and low Schirmer’s test scores.

Variable	Univariate		Model 1		Model 2		Model 3	
	OR (95% CI)	*p* Value	OR (95% CI)	*p* Value	OR (95% CI)	*p* Value	OR (95% CI)	*p* Value
Urine Cd	High OSDI (categorical variable) ^a^							
	2.260 (1.116, 4.578)	0.024 *	2.309 (1.099–4.850)	0.027 *	2.043 (0.953–4.376)	0.067	2.016 (0.959–4.239)	0.064
	Low Schirmer’s Test (categorical variable) ^b^							
	0.849 (0.551–1.308)	0.849	0.655 (0.370–1.159)	0.146	0.594 (0.322–1.094)	0.095	0.597 (0.324–1.103)	0.099

* *p* < 0.05. Model 1: adjusted for age; Model 2: adjusted for age and smoking; Model 3: adjusted for age, smoking, and drinking. Abbreviations: Cd, cadmium; OSDI, ocular surface disease index; OR, odds ratio; CI, confidence interval. ^a^ Cutoff point of OSDI was 6.5 (50th percentile); higher OSDI scores indicate more severe dry eye symptoms. ^b^ Cutoff point of Schirmer’s test was 7 mm (50th percentile); lower Schirmer’s test scores indicate more severe dry eye symptoms.

## Data Availability

Not applicable.
